# The Hospital Nursing Supplement

**Published:** 1894-05-26

**Authors:** 


### "The Hospital" Nursing Mirror.

Being the Extra Nursing Supplement of "The Hospital" Newspaper.

[Contributions for this Supplement should be addressed to the Editor, The Hospital, 428, Strand, London, W.C., and should have the word "Nursing" plainly written in left-hand top corner of the envelope.]

### News from the Nursing World.

### PRINCESS MARY AT SOUTHWARK.

A most useful and excellent institution has been opened by Her Royal Highness the Duchess of Teck. This is an establishment for the use of women and girls employed in shops. It consists of a restaurant, recreation rooms, and rooms for classes and lectures on different subjects. Mr. F. Wigan has given £600, and an anonymous donor £200, towards the necessary expenses.

### NURSES AT WINDSOR.

Her Royal Highness Princess Christian, together with Prince Christian and Princess Victoria of Schleswig-Holstein, were present at a performance given last week at the Windsor Theatre in aid of the Institution of Nurses in Windsor, in which the Princess is so much interested. The programme was an interesting one, consisting of "Ipsithilla," a Greek idyl, and "The Money Spinner." The cast consisted of many well-known ladies and gentlemen.

### A COUNCIL OF HOSPITAL MATRONS.

Miss Isla Stewart, matron of St. Bartholomew's Hospital, is likely to be an active promoter of the scheme to organise a council of hospital matrons for the discussion of their professional work and status. She issued invitations for the 23rd inst. to discuss the proposed organisation.

### NURSES AND COOKING.

How often the excellent services of the district nurse and the doctor are hampered by the wretchedly cooked diet which alone so many poor patients can procure. We are glad to note that nurses themselves are beginning to acknowledge that one very essential accomplishment has to be added to the art of healing, and that one who can superintend or execute a little simple cooking, is an added power and a blessing amongst the poor. Even in rich homes, the nurse has often difficulty in getting the patients' diet suitably cooked, but in the cottage it is next to impossible, and a little help would go a long way in this direction.

### THE WRITERS' CLUB.

Although the Women Writers' Club is still a young institution, it is better established already than some of the older ones. Certainly its recent removal into larger premises is a good proof of the increasing number of members, and the pleasant rooms at Hastings House, Norfolk Street, are likely to be highly appreciated by busy journalists. The new premises were formally opened on Friday, 18th inst., by H.R.H. Princess Christian, the president of the club, who, in a few kindly words expressed her interest in women and their work. Lady Jeune gave a little address on the club, and its objects, its foundation, &c. Amongst those present were the Duchess of Buckingham, Mrs. Humphrey Ward, Madame Bellox, Mrs. Lynn Linton, Mr. and Mrs. George Alexander, Mr. George Burgin, Mr. Zangwill, Mr. Thomas Harding, and many well-known women writers.

### THE NURSE DOLLS.

The Nurse Dolls are now on a visit to Hull. Whilst there, they will appear at the annual sale of work in aid of the Samaritan Fund in connection with the Victoria Hospital for Sick Children, which will be held in the hospital Park Street, on Thursday, May 31st, and Friday, June 1st. The sale will be opened on the first day by the Mayoress of Hull (Mrs. Richardson).

### A SAD LESSON.

Few of those who comment on the fine wards at St. Thomas's Hospital, when they are seen flooded with daylight, and with plenty of workers within easy reach of each other, ever cast a thought to the contrast which night must bring in them. Apparently each of those huge rooms is left in the charge of a solitary nurse, who is practically alone on the floor without any proper means of communicating with her fellows whatever emergency may arise. Of course, this serious drawback in an institution which shelters hundreds of sick folks, can be to a certain extent remedied by electric bells and speaking-tubes, but the formation and size of the wards would seem to make the presence of a second nurse always necessary. A sad tragedy occurred in the hospital last week, a patient committing suicide by drinking poison during the nurse's momentary and unavoidable absence. The coroner's questions at the inquest elicited the fact that poisons kept for testing purposes are left on the table—a dangerous practice which of late years has been confined to a very limited number of institutions. St. Thomas's School has done so much to further improved nursing that doubtless adequate locked-up poison cupboards, and extra night nurses will immediately receive the attention their importance demands.

### IN AID OF TWO CHARITIES.

The Chelsea Town Hall was crowded on Monday, when an entertainment was held in aid of the Grosvenor Hospital for Women and Children and St. Peter's Home for Aged Poor, Vauxhall. The programme was an excellent one, and the platform gay with a belt of flowers with a background of palms. Amongst the artistes who gave their services on the occasion were Mr. Henry B. Irving, who recited "The Gladiators"; Mr. Lionel Brough, who delighted his audience by a series of inimitably told anecdotes; and Mr. Rutland Barrington, who recited "The Burglar's Story." Miss Maggie Gorst danced very gracefully, and the Queen Vocal Quartette of Ladies sang in a very charming manner. Both charities concerned will certainly receive substantial benefit by the performance.

### A NEW HOME FOR NURSES.

Friday last was a gala day at the London Fever Hospital, when Lady Balfour of Burleigh opened the new Home for the nurses in the grounds of the hospital. Fever nursing admits of less variety in the lives of nurses than any other, and it is but right that all reasonable comfort at any rate should be provided for them. At the London Fever Hospital nurses will have no reason to complain in future. The comfortable premises, arranged to accommodate thirty-six nurses, is provided with excellent general sitting-rooms. It is hoped that friends will shortly come forward to provide a library and a piano to afford some recreation for the nurses and make the Home more comfortable. The Home cost £7,000, of which £2,000 still remains unsubscribed.

### AN INTERESTING MEETING.

The Association for the Improvement and Compulsory Registration of Midwives, held a drawing-room meeting on May 18th, at 29, Kensington Square, by kind permission of Mrs. Powney Ebden, Lady Mary Carr-Glynn in the chair. A paper describing the past and present status of midwives and urging on the meeting the necessity of compulsory legislation was read by a delegate from the Midwives' Institute. The medical aspect of the question was ably dealt with by Dr. Annie McCall, and Miss Hughes described in forcible terms the terrible results she so constantly came across, as a superintendent of district nurses, from the employment by the poor of untrained and ignorant midwives. Some real discussion followed, and the meeting showed an earnest and enlightened interest in the question which promises very well for the future. The hon. secretary of the Association is Miss Robinson, 95, Philbeach Gardens, and the associates are ladies who agree in considering some legislation for midwives desirable and promise to use their influence with members of Parliament and other influential persons to forward the passing of some measure which will give that protection to English mothers that is enjoyed in every other European country.

### A PIONEER OF NURSING.

The recent interesting ceremony of unveiling the memorial to Mrs. Wardroper has brought the early days of nurse-training and the name of Florence Nightingale especially before our notice. Mrs. Wardroper was spoken of as Miss Nightingale's "lieutenant" by the Archbishop of Canterbury. Lady Maria Forester, who has just passed from amongst us, might be entitled her "general." She was the first mover in the scheme of which Miss Nightingale became the heroine. To her is given the credit of having found and selected Miss Nightingale for the work, therefore the world owes much to Lady Forester. The discriminating power of selection is a valuable gift, and we must ever be grateful to the memory of one who, through this power, gave us a Florence Nightingale, and thus was through her a pioneer of efficient nursing.

### ROYAL BRITISH NURSES' ASSOCIATION.

At the last meeting of the Executive Committee of this association it was announced that Miss Ravenhill, late lecturer to the National Health Association, had been elected secretary of the association. The offices of the society are now at 17, Old Cavendish Street, W.

### A WISE EXAMPLE.

The King's Norton Board of Guardians is, from a scientific point of view, somewhat unfortunately constituted. At a recent meeting one of its members proudly announced that eight of his fellow guardians were anti-vaccinationists, and he could say that "none of the number had been so foolish as to undergo a silly and foolish rite." This enlightened guardian went on to remark that he was sorry in his youth "he was foolish, ignorant, and stupid enough to be repeatedly vaccinated before inquiring into the matter, but for twenty years past he had been in a more reasonable state of mind." The sarcastic comment of a colleague that it was satisfactory to know that these ardent anti-vaccinationists took the precaution, nevertheless, of undergoing this same "rite" in early youth, was excusable. It was certainly well to postpone inquiries until after revaccination. We hope, for their own sake, the inhabitants of King's Norton will follow the lead and do likewise.

### HEROIC TREATMENT.

Nurses in India meet with many novel experiences, which at any rate equal those in the out-patient departments of English general hospitals. Ink and putty applied to burns by ignorant friends are common incidents here, whilst in India finely-powdered red pottery is applied to inflamed eye, and a patient suffering from enteric fever has strong medicines surreptitiously administered by friends who do not appreciate the enlightened "treatment" of qualified doctors from Europe. It is hardly surprising to hear that the eye application was followed by a perforating ulcer, and that the victim of the fever shortly succumbed to the ignorance of his relations.

### SHORT ITEMS.

An article on "Nurses and their Vocation" is contributed to the *Gentlewoman* for this week by H.R.H. Princess Christian, dealing with the importance of a three years' training for nurses and other aspects of nursing questions.—The Enniscorthy District Nursing Association is making an appeal for gifts of old linen, calico, flannel, and underclothing.—The Basford Board of Guardians have appointed Nurse Robottom to the post of girls' caretaker.—One hundred and twenty patients were tended by the nurses of the Warwick Nursing Association, now affiliated to the Queen's Jubilee Institute, and 3,006 visits paid during the past year.—Two probationary nurses have been appointed to assist the head nurse at the Cardiff Union. A suggestion is on foot to increase the salary of the head nurse from £44 to £50, instead of £46, the ordinary annual increase.—The Bromyard Nursing Association report a satisfactory year's work.—The nurse at the hospital of the Neath Rural Sanitary Authorities has been awarded £3 for excellent services during a small-pox epidemic. It is pleasant to be able to record appreciation of good services.—The nurses of the Bromyard Nursing Association have paid 1,017 visits to patients during the year. The expenditure and income were equally balanced.—The success of the Nursing Institute of the Devon and Exeter Hospital was especially commented upon at the last meeting. It is a source of revenue to the hospital, not a drag on its resources as was feared it would prove by some.—A successful bazaar has been held in aid of the popular Nursing Institution of Limavady, Ireland.—The Honley Nursing Association have closed a useful year of work, with a balance of £33 to the good.—The Ladies' Association for the Care of the Sick Poor, at Blackpool, are entertaining the project of engaging a trained nurse to work for them.

### On General Nursing.

By ROWLAND HUMPHREYS, M.R.C.S., L.R.C.P.Lond.

XIII.—PNEUMONIA (BRONCHO-PNEUMONIA)—*continued.*

THERE are certain diseases in which the acute form is especially likely to supervene, such as measles and the severe form of bronchitis, due to inflammation of the smaller or capillary tubes.

As the first sign the fever rises to 104-105 Fahr., the respirations are greatly increased, and out of proportion to the pulse; the nostrils move in a well-marked manner in proportion to the rate of the respiration; the face, the lips, and often the finger-nails, become bluish; the patient becomes restless, slightly delirious, and the cough becomes more frequent and causes pain.

If the case does not improve, the patient becomes more and more delirious, less and less easy to rouse, and finally passes into a state of coma, from which it is almost impossible to rouse him, and he dies from gradual failure. If the case progresses favourably, the patches of consolidated lung increase in number, the temperature, though keeping high, runs a somewhat irregular course, the cough decreases as the inflammation extends, and there is, as in capillary bronchitis, marked retraction of the lower ribs, these being drawn in by the action of the diaphragm (the great muscle of breathing which divides the cavity of the chest from that of the abdomen), instead of rising and coming forward, as is the case in natural breathing.

The child gradually, almost imperceptibly, improves, and one day one finds the child playing with its toys and half sitting up where there had been little at the previous visit to lead one to expect such progress. The complaint does not entirely end here, however, and its duration cannot be regarded as entirely confined to the one to two weeks during which the symptoms are acute. The lung long shows signs of thickening and deficient powers of aeration, and it is a common thing to find in a child who is having its chest examined as a matter of routine traces of a previous attack of pneumonia, as shown by the resistance to touch, and presence of dulness on percussion, and increase of the powers of the lung for conducting sound, all indicating an inflammatory process which has thickened the lung tissue, and consequently has injured its special powers in respect of the aeration of the blood.

The other form, the more chronic, is a much less marked form in respect to its acute symptoms; it is of gradual development in respect to its signs, and owes its onset to the presence of an acute catarrh, or an attack of pertussis, or other illness which predisposes to it. Its supervention is marked rather by facial change than by any other sign perceptible to bystanders. The child looks tired and blue, and the cough becomes less. The duration of this complaint is marked by the passage of months, and serious damage to the lungs often, indeed usually, results, and the child dies without one'sbeing able to do more than, perhaps, to relieve the severer complications. The causes of death are failure of nutrition, failure of aeration of the blood, and failure of the heart from the additional strain it has to meet in forcing the blood through the compressed and overloaded vessels in the lung, while its muscle has been weakened by its own nourishment following that of the body generally and becoming deficient. The strain on the heart is thrown on its right side, the weaker to begin with, and the least able to develop power to meet the extra strain. The failure in the aeration of the blood appears to be the principal cause of death, though the heart's condition has a great deal to do with it. The author of this paper, some years since, was called out one night to see a child who was said to be dying, having had many attacks of pneumonia, or what was probably the real state of the case, having had a series of exacerbations of temperature as a consequence of the progressive lung disease. He found the child in a state of deep unconsciousness, from which the friends had been unable to rouse it for many hours. After repeated hypodermic injections of brandy it became conscious, and recovered sufficiently to sit up in bed and recognise its mother. It relapsed some hours later, and the friends allowed it to die without sending for medical assistance. He had a similar case not very long after, and is inclined to think that with the help of stimulants to aid the heart and oxygen to assist the deficient powers of the lungs, some considerable advance should be made in the successful management of such cases.

The treatment is to be directed to preventing the onset of broncho-pneumonia in those forms of illness in which there is a tendency to it. Thus in measles the patient should be kept in bed in a warm room, and put into a steam tent on the first sign of bronchial catarrh. In pertussis the cough, which so exhausts the patient, and so strains lungs and heart, should be relieved as much as possible, and for this purpose nothing seems better than the administration of antipyrin by the mouth combined with frequent fumigations of a mixture of equal parts of carbolic acid and conium juice. In diphtheria, in which disease the onset of broncho-pneumonia is almost certainly the forerunner of death, little can be done beyond giving stimulants in sufficient quantity, and, if this has not already been done, putting a steam tent round the child. Poultices, or rubbing with some stimulating liniment, will at times relieve a good deal. In the acute form the temperature often keeps up in the most alarming manner, and should be reduced by the methods mentioned in the last article, such as cold compresses, sponging, or bathing. Attention to the general health is what is principally required with the more chronic form. In both varieties the condition of the heart should be remembered, and all additional strain taken off it by keeping the child in the horizontal position as much as possible, amusing it while insisting on its lying down, and keeping it as far as possible from those attacks of passion which, in some families, seem to be the determining cause of death. Expectorants are needed in most cases, and, if there is much trouble in getting the child to take medicine or if there is much gastric catarrh, apomorphia, an excellent expectorant, may be rubbed in through the skin, and in the same way, in larger doses of the same drug, an emetic may be administered. This is often very useful in clearing the bronchial tubes of accumulated mucus, just as in phthisis, where the morning sickness clears the tubes of the mucus which has accumulated in them during the night.

In many of the acute or sub-acute cases it is very difficult to distinguish between the disease we are treating of and tubercular meningitis, and in many cases only the result of the disease shows its true cause. The same may be said of acute miliary tuberculosis, where the lung becomes studded with little solid masses of tubercle. This last disease, a very rare one, is rapidly fatal. It is often difficult to determine the position of the patch of inflamed lung, where deeply situated, which causes the symptoms of the disease. The author had one such case in a boy quite recently, who had been the subject of the complaint previously, and who, in this last attack, presented no local sign to show what part of the lung was affected, the disease being principally indicated by other signs, such as increased respiration, movement of the nostrils, &c.

*Chronic Pneumonia*, also known as cirrhosis of the lung and fibroid pneumonia, is a condition of slow contraction of the lung, caused (1) by some chronic irritant, as occurs in trades where fine particles of dust are being constantly inhaled; (2) by some condition of the lung, in which after an attack of acute pneumonia or abscess of the lung, it passes into a chronic inflammatory state; (3) by broncho-pneumonia as already stated; (4) by changes taking place in a lung which has long been compressed by pleuritic effusion. As a result of these changes the breathing power of the patient becomes less and less, the strain thrown on the heart greater and greater, and he finally dies, after a course of, perhaps, ten or fifteen years, from cardiac or respiratory failure.

### Women's Work.

### WOMEN IN THE CIVIL SERVICE.

One of the many movements which has of late years tended to enlarge the sphere of their work and usefulness has been the admission of women to the clerical staff of the Civil Service. They have taken and retained their places there quietly and unobtrusively, and by their care, accuracy, and reliability have very considerably raised the standard of value in which women and women's work are held. In fact, they have been so great a success that every year more of them are admitted to posts of different degrees of importance and responsibility.

A pleasant feeling of comfort and security is enjoyed by those who work under Government, and taking into consideration the hours, chances of promotion, and various other advantages, it is by no means surprising to find how keen is the competition.

This article is intended to be a short account of the different branches of the Civil Service, with a little information as to how appointments may be obtained, the study necessary, the approximate salaries, and the work to be performed.

The three chief departments of the Post Office are those of the telegraph learners, the female sorters, and the female clerks; and as entrance is almost always obtained by examinations, it is open to anyone who can satisfy the primary requirements as to age, character, and health to compete.

To begin first with the telegraph learners. The examinations are held twice a year, and the dates can generally be found in the advertisement columns of a daily paper or in the *London Gazette.* Candidates entering for this examination must not be under fifteen years of age or over eighteen. The subjects are not very alarming, but the standard is rather high—writing from dictation, handwriting (which must be clear and legible), orthography, English composition, arithmetic, and geography. A good and very helpful idea is to get a set of the papers which have been sent in for these examinations; these may be procured from Messrs. Eyre and Spottiswoode, East Harding Street, Fetter Lane, and they are specially useful in the case of composition, arithmetic, and geography. It is necessary to pass in every subject, but there is nothing to prevent any girl from making several efforts as long as she is within the prescribed age. After having passed the examination, the candidate is shortly called upon to enter on a period of instruction, lasting about three months, in the Telegraph School, attached to the General Post Office; there she receives a free course of instruction in telegraphy, and is initiated into the mysteries of receiving and despatching messages; but if, at the end of a month or a later period, she shows no aptitute for the work of a telegraphist, her nomination is cancelled. On the other hand, if at the end of three months she has displayed sufficient aptitude and receives a certificate of proficiency from the school, she begins her probationary period at a commencing salary of 10s. a week, which is gradually raised to 12s. and then to 14s., and from this point increases by 1s. 6d. a week up to 30s.; but it is not necessary to stop there, for she may use her position of telegraph clerk as a stepping-stone to either a "sortership" or a "clerkship." Telegraph learners are liable to be called upon to do Sunday work, but it is very seldom indeed that this liability is enforced.

In the "female sorterships," the limits as to age are the same as with telegraph learners, but in this case we have to consider the question of height, as no girl is eligible who is under 4 feet 10 inches. This explains itself when we know that the work is connected with the payment and issuing of money orders, and the sorting and arranging of official papers at desks of a fixed height. The subjects of the examination here are slightly different, viz., arithmetic (first four rules, simple and compound), the geography of the United Kingdom, handwriting, orthography, and, finally, what perhaps the most troublesome of all, the reading and making fair copies of badly written, or illegible MSS. The hours in this section of the Post Office are about the average, from eight in the morning to five o'clock in the evening, with an interval of half an hour for luncheon. The salaries in this branch begin at 12s. a week, rising after the first year to 13s., after the second to 14s., and then it goes on with a rise of 1s. 6d. a year to 21s. 6d., and yet further promotion can be obtained up to 30s. a week. "Sorterships" are always in great request, as they also can be used as a stepping-stone to a "female clerkship," and naturally the experience gained as a "sorter" helps greatly in the preparation for the more difficult examination.

The last branch of the Civil Service to which a girl has access is that of the "female clerkships." The subjects for this examination are similar to those in the other branches; the geography and history cover a much larger area; the English composition is also an important point, and, above all, speed and correctness with regard to money and figures is of supreme importance in this examination. The limits of age in this branch are between 18 and 20 years, and the examination standard being so high, the candidates usually are of a somewhat higher social status. The salaries commence at £65 a year, increasing £3 yearly up to a £100, and beyond that there are some few posts at £180, the promotion to which, of course, depends on special aptitude and merit. The hours are from ten a.m. to five p.m., with, of course, an interval for lunch.

The best means of preparation for the Civil Service is to join classes specially devoted to coaching candidates for these appointments. There are plenty of such classes in existence with excellent records of past successes, and information with regard to the regulations, &c., can always be obtained from the Civil Service Commissioners.

Successful candidates for any branch of the Post Office have to go through a very searching examination by Miss Edith Shove, M.D., as to their health and physical soundness, before they are appointed, and it is well to know that as well as the general Bank Holidays a month is given every year. There is a regulation that all lady clerks have to resign their appointments on marriage, and, in consequence, the promotion is much quicker than it otherwise would be.

Mr. Fawcett rendered many services to the nation, and it was he who first insisted on the extended employment of women in the Post Office, and it is good to know that the result appears to have answered in every way his expectations.

A. A.

### Novelties for Nurses.

We have received a new catalogue for 1894 from the Cellular Clothing Company, Limited, which is worth study by those who desire to obtain a maximum of comfort in their underwear. Every variety of undergarment can be had in this pleasant material, and special shapes for those who desire them can be made in any of the fabrics in two or three weeks. We note that in some instances the price of the garments has been reduced. The London agents of the firm are Messrs. Robert Scott, 15, Poultry, Cheapside, E.C., and Oliver Bros., 417, Oxford Street, W., from whom all particulars may be procured.

### Wants and Workers.

If any nurse would like the weekly number of The Hospital free until November next, Sister C., Barracton Post Office, Cork, Ireland, would be happy to send it to her.

### A Visit to Canada.

By Sister Norma.

### II.—THE SULPHUR SPRINGS OF BANFF.

We arrived at Banff Station at half-past six a.m., at an elevation of 4,500 feet. The mornings and evenings are always chilly, and even in the mid-day sun the heat is never oppressive, for the cordon of mountains, another 5,000 feet above you, send down cool breezes from their glaciers. The Canadian Pacific Hotel, which we thoroughly enjoyed until we saw our first bill, is one of the most typical demands of this luxurious country. There, in the heart of the Rocky Mountains, you are supplied with electric light, telephones, hot sulphur swimming baths, ball-rooms, billiard tables, saddle horses, carriages, and every luxury an Astor or Vanderbilt could desire. It is a magnificent building, with balconies all round it on every storey, cleverly-devised sun galleries project from all the gables, which are more like seventeen than seven, full of rare exotics and comfortable lounges and rocking-chairs. Every balcony and almost every window commands a sweeping view of the beautiful Bow Valley, with its sky-blue river and a perfect sea of tumbled Alps beyond. It was certainly the most health-giving spot I was ever in, and even in one week we presented very different appearances from the pallid dwellers on the plains.

So far we had only tried the private sulphur baths belonging to the hotel, which cost 75 cents each. They were well worth the money to us, but not half so inviting as the beautiful Government baths—one in a natural grotto with a beehive roof, the other clear as crystal in the elbow of the mountain side. Mrs. C. was now feeling well enough to go in for less luxurious surroundings, so we changed our expensive quarters for less attractive ones in the Sanatorium further down the valley, but nearer the station and the town of Banff, which consists of a few "hotels" and chemists, a post-office, and the national park. We were very comfortable, and the place was charmingly clean, and everything was just half the price. The Government baths are a delightful institution; there are two of them, used alternately by ladies and gentlemen; formerly both sexes bathed together as they do all over Canada, but this had to be stopped when the large mines were opened in the district, and the ample wages of the emigrant miners supplied them with the luxuries of sulphur baths.

Of the two, the cave and the open basin, I preferred the latter; it was a beautiful clear pool with a sandy bottom sheltered in the elbow of a hill with big, hot sulphur springs bubbling up into it about eight feet deep. The natural rocky wells round the three parts of it were hanging with queer sulphur formations in stalactites and stalagmites, and everything was fossilized. It was quite big enough for a good swim, and such delicious warmth, about 80 Fah., the air felt clear and crisp in comparison. A diving board formed a sort of balcony for the dressing rooms, which completed the walls of the circle. For 25 cents you were supplied with a bathing dress and towels and a clean and comfortable dressing-room, and the help of a most excellent caretaker. She took the greatest interest in her patients, though her real duties were to keep the place clean and tidy. She was a pretty, happy Irishwoman, and was a decided attraction to the baths. She had a marvellous memory for all her customers' real or fancied complaints. Mrs. C. was a splendid swimmer, though for a while she could hardly use her arms freely enough to enjoy the exertion; she had been a martyr to rheumatism ever since she had lived in Canada. At the end of the first month, however, it had almost disappeared. What fun we used to have; a large party of us from the Sanatorium used to bathe at the same hour every morning in the soft, invigorating water, there was no fear of turning blue at the lips, no necessity for dipping your head under as sea-bathers are always prescribed to do, and you could stay in for an hour without feeling chilly. I had never indulged in sulphur baths before, and the terrible smell at first so disgusted me that I rather scorned the idea of joining the others. For miles round the base of Sulphur Mountain this awful smell seems to follow you, the air is full of it, but once you have plunged into the body of steaming water the smell completely disappears. It is a pretty sight to see people swimming in sulphur water, it has such a lovely effect on the skin. We often spent the greater part of the morning in the baths towards the end of our visit, and we always took just one more plunge when we remembered our dried up home on the prairies. I enjoyed milk and cakes when I came out. Mrs. C. continued to drink the strong sulphur water from a little bubbling stream spurting out from the mountain side. This bath is open from half-past ten a.m. to half-past twelve for ladies, and the one in the cave from twelve a.m. to half-past one. Ladies generally prefer the open basin, it is so much more convenient dressing and undressing in the comfortable rooms with just one step out on to the balcony and then a plunge. The cave is, of course, far the most wonderful. One walks along with lighted torches through a long, narrow stalactited passage, and suddenly emerges into an exquisite dome-shaped cave 40 feet across full of jobbling steaming water; it is only very dimly lit by a natural hole in the roof; in shape it is like the inside of a beehive; there is a plank a few feet wide all round the basin where you can rest and lounge about, for the atmosphere is as warm as a Turkish bath; it is rather warmer even than the water from the great body of evaporated steam. There are no dressing-rooms here, and one almost melts with heat before one can get one's clothes off. There is more room for a good swim here, and men generally seem to prefer the voluptuous air of the cave to the fresh air of the basin; and there is a weird charm in the dome overhead hanging with fantastic sulphur draperies.

The country for miles round is rich in sulphur springs; both the large hotels have their private spring baths, and there have been new public baths opened since I was there which promised to rival even the Government baths in beauty and comfort. There are the "Higher Springs" in the side of Sulphur Mountain some way up, but these are not so much used except by those who are very bad. The higher springs have many tokens of their healing properties; the sight reminded me of one of the pilgrimage spots in the Alps I once visited where a black Virgin is supposed to cure all maladies the flesh is heir to. The walls of her shrine were completely covered with crutches and spectacles and models of legs, ears, arms, &c., thank offerings from the faithful for the cures she had wrought. The poor lady of Einsedelen was certainly black but not comely.

At the different resting places on the mountain by the side of the sulphur stream there is always an eager attendant ready to offer the water, and you are encouraged to drink, pay, and believe by the sight of many crutches hanging up, with the date of the complete cure and the signature and address of the patient, and other touching advertisements for the health-giving properties of the sulphur waters. These higher springs are very picturesque, they run down through the steep well-wooded mountain side, which is covered with queer uncanny plants and strange water grasses, a luxurious and unnatural vegetation seems to flourish and take life in these sulphur marshes. I should have mentioned that patients suffering from any skin complaints were not admitted into the Government public baths; on this point the authorities were rightly very particular, even to the extent of making you show your doctor's certificate.

I should have liked to say something about the sights of Banff; the drive to the deep, dark Devil's Lake (where they catch the 50lb. trout) by the side of the exquisite little canyon, and through a forest so strewn by fire and tempest that it reminds one of Ezekiel's valley of the dry bones; the paddle up the creek to the shallow, sedgy Vermilion Lakes, with their ring of mountain and forest, and swarms of waterfall; the mighty cascade of the Bow River; the Hoodoos or White Friars, those queer natural monuments of hard conglomerate left when the softer rocks were washed away on the benches over the river; and the marvellous contour of the Rocky Mountains—so felicitously named. Banff is 5,000 feet up in the Rockies, and has been described as the Rocky Mountains made easy for invalids.

### On Lecturing.

By One Who Has Done It.

Occasionally they are modestly called "talks" or "chats," at other times the dignified term of "lecture" is freely bestowed. Whatever the title the matter which composes the discourse has for its object the enlightenment of the ignorant. But as all ignorance is relative, all teaching shoul l therefore be intelligently graduated.

To talk of "the circulation" to hearers who are already puzzled by the strange lecturer's voice and manner cannot be deemed deep wisdom supposing the audience to be composed of uneducated people.

When the audience is a distinctly homely one it is, above all things, needful to speak in slow and plain Queen's English. Unluckily, a quick thinker is apt to be a rapid talker, and a rapid talker is not a good rural lecturer. Simple language means a simple and forcible adaptation of everyday expressions, but not weak little sentences culled from a child's "Easy Reading Book." Women should never be addressed like ignorant, innocent children; they need a stronger, more suitable, diet, as the cleverest lecturers know.

If ladies attempting to instruct their poor neighbours would "put themselves in their places" all the difficulties would disappear, and the classes would really be useful, as they are of course intended to be.

It is no good to start only from a standpoint of hospital training, nor is it fair to the hearers. If the speaker would only remember how nursing looked to her eyes before she herself was a learner, she might make a better teacher. The lectures or talks should certainly, so to speak, start from the cottage hearths, and the daily surroundings of the girls and women should be specially considered.

One very practical and popular nurse has sometimes invited the people to bring all their own materials for her to lecture on, and excellent were the poultices, fomentations, and other outward applications which were thereupon produced with but slight supervision. Another lady of much experience found it far easier to instruct mothers on the care of babies delicate spines, when she had shown them an actual specimen of the vertebræ in question. She was too wise to burden their unaccustomed memories with technical terms, but she laid plainly before their eyes the evils which spring from want of knowledge of the proper treatment of children's bones.

If clever women are sometimes bad teachers, assuredly stupid ones must always fail! A lady once stood upon a village platform and discoursed on many things to the surprise, if not the edification of her hearers, for she told them that the blood in the human body was contained in two bags, one was for the good blood, the other for the bad, and these were called the lungs. She concluded, "You've often heard of persons living with only one lung, my good people? Yes! Well, it's quite possible for people to live for years with only one lung." "Dear me," said one of the audience, "does it matter whether 'tis the bag of bad blood or the other?" The lecturer became conveniently deaf, for the question went beyond her ordinary daily catechism.

Surely those who aspire to teach the first laws of health should carefully study them beforehand, otherwise the old parable of a blind leader of the blind will be disastrously exemplified. And again it should be borne well in mind that no one can pass on knowledge satisfactorily of which she has not proved the truth.

When nursing is specially in question, the audience want to learn how best to tend their own sick folks with the means already at their command. They do not need treatises on disease or details of varied treatment. They simply want to know the best thing to do, and the best way to do it, in every emergency, whether it be a small or a great one. And this applies to many audiences besides rural ones.

Practical demonstrations of certain nursing points are generally highly appreciated, and certainly when men also are admitted to these they are most attentive and observant. The old and ridiculous notion that useful arts are beneath the dignity of the male sex is luckily almost exploded, and we find neatness and dexterity in men growing yearly greater. Surely no man is the worse for being able to wring out a fomentation?

### The Probationer's Night Watch.

Weary all my limbs, my head is aching
As I sit, a moment, watchfully,
And I mark each restless bed, betraying
Wakeful pain, which keeps me company.

Silent lies each head upon the pillow,
No faint voice calls, half-ashamed, for me,
Easier far to tender the slight service
Than to listen, waiting powerlessly.

Powerless 'mid this crowded, wakeful stillness,
Where each soul is tossed with separate pains,
And the despairing sum of human anguish
Brooding o'er my heart, like lead, remains.

Colder grows my heart, in bitter musing
On the cureless, soundless griefs around,
That not sinners only, but the guiltless,
'Mid the hapless sufferers' ranks are found.

Why should these, the helpless, bear the burden?
Why the mother from her home be torn?
Why upon the little child be laden
Pangs which by the hero scarce are borne?

Where is Love, which should direct the courses
Of relentless Nature, sternly strong?
Where that Power and Wisdom by which justice
Reigning o'er the earth should right the wrong?

Softly through the ward there steals a movement,
As the strained dumb stillness dies away,
Sweetly, faintly on the night wind hasting
Come the midnight chimes of Christmas Day.

Eagerly the faces turn to hearken,
Sorrow, want, and anxious boding fear
Fade a moment at the Master's message,
"Christ is born, fear not, His peace is here."

'Ere the ringing dies away in silence
And the long night watch begins anew,
From my faithless heart the bitter doubting
Dies abashed before the message true.

Born upon this night, He came to show men
Highest love in deepest pain concealed;
Stainless lived and suffered that His children,
Suffering too, should taste His love revealed.

Wise Physician, from the seething poison
Of disease and failure, want and strife,
Stooping low, He has distilled th' elixir
Whoso drinks shall taste eternal life.

Nevermore we need to ponder blindly,
O'er the unfathomed mystery of pain,
All we care to know is in the tidings
Christ is born, He turns our loss to gain.

### Our Examinations.

We regret that no answer received to our April question was sufficiently good to merit a prize. We hope the one set for May will be fully answered by a large number of our readers.

Question for May.

How would you give first aid to a person who had been badly scalded or burnt?

Answers, which should be written on one side of the paper only, to be sent on May 31st, to "Nursing," Editor of The Hospital, 428, Strand, accompanied by the full name and address of the writers.

### The Nursing of Army Sisters.

By A. O. Grey.

III.

The life of an Army Sister at Netley ought to be an ideal one; the work is most interesting, and the surroundings lovely. The order to proceed to a station hospital is usually received with regret; the unknown is so full of uncertainty, friends must be left behind, and a fresh start made. The system of working is now obviously different. There are only three sisters instead of the larger staff at Netley, and as there is no accommodation in hospital, quarters are provided, generally at some little distance, where the nursing sisters and servant are under the superintendence of a senior. The "Acting Superintending Sister" undertakes the housekeeping and catering, and if she is a good manager, the sisters live very comfortably on the allowance given by Government. Having her ward duties the same as the juniors, it is not always easy for her to market to the best advantage; in the event of her having a run of bad cases, it may be almost impossible for her to leave the hospital.

It will be readily understood that nursing in a station hospital presents many difficulties, some garrisons being much larger than others; the work is consequently heavier, and the sister rarely feels off duty when there are bad cases in her wards. There is no night sister, and although orders are written and thoroughly explained to the orderlies, there is always a feeling of uncertainty as to whether they will be fully and efficiently carried out. Again, each sister takes it in turn to be on duty during the afternoon; thus being on duty every third day for the entire day, and responsible for her own and the others' patients.

The present Director-General is not in favour of amusement or relaxation for the nursing sisters. Instead of being encouraged to secure rest of mind and body by indulging in an occasional visit to the theatre, or concert, as is the case in civil hospitals, where tickets are often sent to the nursing staff, the sisters are not allowed so to employ their hours off duty without asking the permission of the medical officer in charge. So much for the false idea that prevails regarding the "good times" in military nursing.

Much has been said about the position of the A. N. S. The sisters receive the allowances, &c., of a staff-lieutenant, but the considerations due to that rank are not always theirs. From most of the medical staff there is the greatest of courtesy and kindness experienced, but there are still some of the old school of surgeons who do not seem to be able to disassociate the "Sairy Gamps" of their hospital career with the more advanced and refined style of the present day nursing, and to work under whom can neither be a privilege nor a pleasure. As regards the outside world, the sisters' social position is just what they choose to make it. It is in their power to make friends of the nicest people in the garrison, and it is entirely the fault of the sisters themselves that in some stations they are not treated with the same *esprit de corps* and hospitality that they expect.

Hopes have been entertained that the retiring age might be reconsidered, so that the full pension, which at present is only paid when the sister is sixty, might, under some circumstances, be granted sooner. A sister who has done a large share of foreign service and campaigning is naturally prematurely aged and less able for work than those who have not had such active duty. It seems only reasonable that if she finds herself at fifty unfit to bear the burden and heat of the day, although not actually disabled, she might claim the hardly-earned pension, and have a few years of the rest she has worked so hard for. If the retiring age were optional at fifty, Government would confer a great boon on its nursing sisterhood.

Should a sister be so unfortunate as to be on the sick list—and who is proof against illness?—she will find a marked difference between the treatment she would receive in civil hospital and that she has from Government. There is always difficulty in getting a substitute. Besides having the extra home nursing the two remaining sisters must do the whole of the hospital work as best they can. If help is sent it is generally when the worst is over, and the sister, who has, it may be, had an attack of influenza, must hurry on duty as soon as possible, otherwise she loses the thirty days' annual leave which is to build up her health for the ensuing year. It would seem that sickness is looked upon as a sort of recreation; every day so spent is deducted from the aforesaid thirty days, and the sister must either do without leave or take it without pay, unless she is granted special sick leave, which is necessary after such illnesses as enteric or malarial fevers. This is obtained from the Commander-in-Chief through the medium of the medical officers. These rules do not apply to the staff-lieutenants, with whom the sisters are supposed to rank; they are exempt from having their days of sickness deducted from their leave. Perhaps there may come a time when the weaker sex will have equal consideration.

Military nursing, on the whole, will be found an excellent experience, and a few years of its economies and discipline are most useful training for those who aspire to the higher appointments in the nursing world.

### Nursing in Ireland.

By Our Irish Correspondent.

Nurse J. Daly, formerly on the private nursing staff connected with Dr. Steevens' Hospital, Dublin, has just met with a piece of good fortune. She has been nursing a private patient named Warren for the past two years. A few days ago he died, leaving her, in consideration of her care and skilful attendance during his long and wearisome illness, an annuity of one hundred pounds.

The Jubilee Concert got up by Mrs. J. B. Story, in aid of St. Mark's Ophthalmic Hospital, has been an unqualified success. Miss Hime (niece of Dr. Maurice Hime, the well-known master of Foyle College, Londonderry) came over to Dublin to perform at it. She is a magnificent pianist, being one of the favourite pupils of Madame Schumann, and an Associate of the Royal College of Music. St. Mark's Hospital was established by the late Sir William Wilde (then Mr. Wilde), at his own expense, in 1844. At present it contains 50 beds, the number of indoor patients yearly averaging 700, and the dispensary patients 4,000. One hundred and fifteen pounds was realised by the concert in aid of its funds.

Another pleasant and successful concert was recently given in the Assembly Rooms in aid of the Queenstown Hospital. It was under the patronage of Rear-Admiral St. John, R.N., and Colonel Kingscote. The violin playing of the Misses St. John was heartily applauded.

Dr. O'Connor, of Clane, co. Kildare, was last week the recipient of a most complimentary address and testimonial from the people of the dispensary district, where he has lived and worked for some years. He has gone to Celbridge, and is succeeded in Clane by Dr. W. Coady, who belongs to a well-known Kildare family. Dr. O'Connor's popularity is shown by the signatures attached to his address, which comprise names of representatives of all classes, religions, and opinions.

The popularity in Dublin of Surgeon-Major-General Collis, late P.M.O. in Ireland, was evinced by the general regard expressed when it became known that he was about to retire under the age clause. The officers of the medical staff in Ireland entertained him at a farewell dinner, at which a large number of influential military men of all grades were present. At this banquet a gratifying tribute was paid by him to army nurses. He said, in reference to the work done by the Army Nursing Sisters: "When first these ladies were introduced the feelings of officers and men as to their utility were mixed; but there is now no doubt as to their value. Nursing is a most important part of treatment, and although it would be absurd to suppose that nursing without treatment would prove effective, treatment without proper nursing is as bad. Our sisters do their duty faithfully, and their influence in brightening a ward and cheering a sick man is now an evident fact in all our greater hospitals, and the soldier is very grateful for it," which is high praise from such a distinguished authority.

Mrs. Beaumont-Nesbitt, of Tubberdaly, near Philipstown, King's County, is trying to organise a District Nursing Association in that neighbourhood. It is very badly required, there being no hospital in the vicinity. The King's County Infirmary at Tullamore is about sixteen miles away from the Dispensary at Rhode, and the number of beds there, and kind of cases admitted, exclude the class of patients for which district nursing is most to be desired.

Miss Eames, daughter of Lieutenant-Colonel Eames, has been appointed Head Nurse in St. Mark's Ophthalmic Hospital in the place of Miss A. F. Hartford, lately appointed Matron to the King's County Infirmary, Tullamore.

It is rumoured in nursing circles that the very popular Lady Superintendent of the Adelaide Hospital, Dublin, thinks of resigning her post. Everybody connected with the hospital must regret this, as Miss Poole has occupied the position for several years, and has been the means of vastly improving the status of the institution. Miss Poole is the sister of Dr. Poole, a well-known physician practising in Belfast. Another of her brothers has for a long time held the appointment of Bursar in Trinity College, Dublin. The Adelaide Hospital is the sole exclusively Protestant institution in Dublin. It is chiefly supported by voluntary contributions.

A three-volume novel is now in the press, which will probably create some interest, from the fact that it is written by an Irish nurse. "Stepping Stones" will, it is to be hoped, prove a success. The authoress is a well-known contributor to several of our home journals and magazines, although this is her first book.

### Notes and Queries.

The contents of the Editor's Letter-box have now reached such unwieldy proportions that it has become necessary to establish a hard and fast rule regarding Answers to Correspondents. In future, all questions requiring replies will continue to be answered in this column without any fee. If an answer is required by letter, a fee of half-a-crown must be enclosed with the note containing the enquiry. We are always pleased to help our numerous correspondents to the fullest extent, and we can trust them to sympathise in the overwhelming amount of writing which makes the new rules a necessity. Every communication must be accompanied by the writer's name and address, otherwise it will receive no attention.

### Queries.

(65) *Ireland.*—Would a nurse be taken as assistant stewardess for one voyage to America or elsewhere, and where should I apply about same? —*Stewardess.*

(66) *Midwifery.*—Can you tell me of a good infirmary in London, or South of England, where midwifery is taught in return for a few months' service?—*Nurse S.*

(67) *District Nursing.*—Please give addresses of training schools for district nurses in London and the country.—*Jenny Lind.*

(68) *Switzerland.*—Would it be worth while for a certificated nurse to go to a Swiss health resort on the chance of work?—*D. F.*

(69) *"Braloo" Window.*—Can you tell me where I can get information concerning this and other windows constructed so as to lessen the dangers of cleaning?—*J. C. S.*

### Answers.

(65) *Stewardess (Ireland).*—Write to secretaries White Star Line, 34, Leadenhall Street; Cunard Company, 13, Pall Mall, S.W.; and Guion Line, 7, Pall Mall, S.W.

(66) *Midwifery (Nurse S.).*—We do not know of any such institution.

(67) *District Nursing (Jenny Lind).*—Metropolitan and National Nursing Association, 23, Bloomsbury Square; Queen's Jubilee Institute; St. Katherine's Hospital, Regent's Park; and Birmingham District Nursing Society, 98, Newhall Street. For many others, see "Burdett's Hospital and Charities Annual," Scientific Press, 428, Strand, W.C.

(68) *Switzerland (D. F.).*—We are making inquiries for you as to certain districts. Look for answer later.

(69) *"Braloo" Window (J. C. S.).*—The Hospital for June 3rd, 1893, contains description of Braloo window; other windows are fully described in The Hospital for March 17th and 24th, 1894.

### For Reading to the Sick.

### CONVALESCENCE.

**Motto.**

Whether I live, I live unto the Lord, or whether I die, I die unto the Lord. —*St. Paul.*

**Verses.**

The day is over, the feverish careful day;
Can I recover strength that has ebbed away?
Can even sleep such freshness give, that I again
Should wish to live?

Let me lie down! No more I seek to have
A heavenly crown; give me a quiet grave;
Release and not reward I ask—too hard for me
Life's daily task. —*T. T. Lynch.*

Not now my child!—a little more rough tossing,
A little longer on the billows' foam,
A few more journeyings in the desert darkness—
And then the sunshine of thy Father's home.—*C. P.*

Teach me to live! 'Tis easier far to die
Gently and silently to pass away. . . .
Teach me that harder lesson, how to live,
To serve Thee in the darkest paths of life;
Arm me for conflict now, fresh vigour give,
And make me more than conqueror in the strife.
Teach me to live Thy purpose to fulfil. . . .
Closer round Thee my heart's affections twine!
Teach me to live and find my life in Thee,
Looking from earth and earthly things away. . . .
Waiting with cheerful patience till Thy call
Summons my spirit to her heavenly home.

**Reading.**

Convalescence is a condition so closely allied to sickness that few persons have attempted to write anything specially adapted to its wants, though there are even more temptations and trials than in confirmed sickness. . . . Some learn how to bear the trials of convalescence better than others. They are naturally of a contented mind, but all have to be watchful, for God intends convalescence to teach those lessons which sickness did not accomplish. Accept patiently your state of weakness; God will only send you just the trials you need to mould and perfect you. . . . He calls you for a time into closer communion with Him. Condemned to be quiet for the good of your body, you have strength enough to worship God in your weakness, and He will teach you some great lessons if you will only accept His teaching. . . Now is your opportunity. Consecrate yourself anew to God in the solemn pause, whilst you are waiting for a renewal of strength, before you again enter into active life with its temptations and distractions. . . . In health and happiness we thought not much of God or eternity; life and its interests absorbed most of our thoughts; there was little or no room or time for God; but now, in the quiet hours of convalescence, we see how in the past we have neglected the means of grace, and, thanking Him for bringing us apart, for saving our life, we resolve not to be among the number of those who, after their recovery, forget God, to Whom they owe it, but determine so to use our quiet hours that we may be numbered with those who, having been healed, return from all their evil ways to give glory to God. . . . Remember, that having been rescued from the very brink of the grave, such a deliverance must never be received in vain. Look upon yourself as brought back into the world for the world's good, amongst men for men's good; by the mercy of God, for the glory of God. Cast away, without hesitation, that selfish, empty life, from which you have been roused by the touch of suffering, and if your complete restoration be long delayed, long to say with the Apostle, "Whether we live, we live unto the Lord, or whether we die, we die unto the Lord; whether we live, therefore, or die, we are the Lord's."—*Adapted from "The Trials of Convalescence."*

☞ For The Muses' Looking Glass, Everybody's Opinion, Book World for Women and Nurses, &c., see page lxxix. et seq.

### The Muses' Looking Glass.

"MARCELLA."*

The story of Marcella Boyce, as Mrs. Humphrey Ward tells it, is, after all, pretty much a story of everyday life, for it is in reality little else than the history of a girl with an opinion. In 1894 all girls have opinions. Marcella's was a political one—tediously, earnestly political; and though it appears at first to have dominated in no small measure Miss Boyce's being, it is barely of equally absorbing interest to outsiders, but that is doubtless the fault of the reader more than of the writer. We have become so immured to the shorter form of narrative, that Mrs. Humphrey Ward's more conscientious, laborious consistency in giving us detailed narrative is shown at a disadvantage. In the rush and tear of life we have few disconnected hours in which to take up a book which loses by being skipped and hurried through, and in her present novel Mrs. Humphrey Ward not only demands our leisure but our minds—a demand which finds its own reward in the perusal of the three volumes under discussion.

Those tempting "Short Stories," which ask so little of our minds, and to which we owe the whiling away of many an odd half hour or so, have done this for us—they have unconsciously created a false measure in our minds. Literature—romance literature anyhow—which is not thus abbreviated, appears to assume exaggerated proportions. This is particularly the case in Mrs. Humphrey Ward's last book. Marcella's history is told with a pre-Raphaelite regard to the value of detail—is here and again sublimated, however, with a good general breadth of treatment. The opening chapters find the heroine in the ancestral domains of the Boyce family. Before this, however, the girl had been thrown with some London friends, who had infected her with their political tenets. These are of the socialistic order, and when we first make Marcella's acquaintance it is as a champion of the poor man's rights, her mission being to redress their wrongs. This sympathy with their sufferings and pity for their poverty dominates the girl's whole being with a withering intolerance of injustice, born of youthful years. Her father's tenants offer Marcella an immediate field of operations, and in this devoting of herself to impersonal causes, ends not her own, lies at once the strength and the weakness of her character. In this living up to a high standing in one direction, many another grievous charge may be laid to the door of this fair aspirant.

Marcella, as we have said, was political. Her own friends suffered through her politics. Blind to all other obligations, blind to all duties of home and society, the girl sees only, with an exaggerated vision, the down-treading of the one class by the other. Other calls do not appeal in any forcible manner to her higher nature; and as an only child, she is barely what one would describe as a dutiful one. But then, of course, heroes and heroines are not called upon to be heroic in all directions, and in the case of Marcella Boyce some sacrifice must be demanded of her surroundings. This appears to be consistent with Socialism. A clear insight into the workings of the girl's mind is given us by her biographer on the occasion of a great ball at a great house, where Marcella is presented to the neighbourhood as the bride elect of Aldous Raeburn, *the* "parti" of the county. On this occasion she is described as standing at the head of the ball-room, "signs of thought and storm," as the writer says, in her face; in her mind angry thoughts warring against her surrounders. "She knew indeed that a great many of these men, who came and spoke to her, were doing their best according to their lights, that improvements were going on, that times were mending. But there were abuses still, and the abuses were far more vividly present to her than the improvements. In general the people who thronged those splendid rooms were to her merely the incompetent members of a useless class. The Nation would do away with them in time" . . . . "and once or twice she thought passionately of Minta Hurd washing and mending all day in the damp cottage; or of the Pattons in the Parish Rooms, thankful after 60 years of toil for a hovel where the rain came through the thatch, and where the smoke choked you, unless, with a thermometer below freezing point, you opened the door to the blast. Why should THESE people have all the gay clothes, the flowers, the jewels, the delicate food—all the delight and all the leisure—and those—nothing? Her soul rose against what she saw as she stood there going through her part."

Later on her "soul" rose still further against her own class, and Aldous Raeburn's love is thrown aside, and the lover finds himself discarded a month before their wedding day. And this, purely on account of a difference of opinion between the two regarding a poacher *protegé* of Marcella's. "'Don't you see, don't you understand?' she explains the day when she breaks with her *fiancé*, 'if we *could* take such different views of a case—if it could divide us so deeply—what chance would there be if we were married? I ought never, never to have said 'yes' to you. . . The poor have come to mean to me the only people who really live and really suffer; I must live with them, work for them, find out what I can do for them. You must give me up, you must, indeed.'"

And Aldous Raeburn accepted her words in better part than perhaps ought to have been expected, especially when his injury was further augmented by the knowledge, learnt from her own lips, that another man had come between them. The "other man" whom Marcella described as having come between them, had appealed to the girl's impressionable nature, and had carried her away by his advanced political views, his schemes, and his speeches. But the reader's sympathies remain with Aldous Raeburn. Now the scene changes, and Marcella leaves her home and joins the great nursing world in London. Here she is thrown again with Mr. Wharton, M.P., Socialist Reformer, the man who had come between her and her lover. Though now free, Marcella refuses his offer of marriage, in spite of the fact that she had never seriously discountenanced his attentions. The third volume sees our heroine reinstated in sole sovereignty in her own domains, untramelled now, and free to carry out her former schemes of the betterment of her tenants, her's now, since her father's death.

But it is characteristic of Miss Boyce that here again her inconsistency evinces itself, and all her purposes and aims are submerged in the return of her affections for her former lover. It is she, herself, who pleads for the continuance of their former relations, and who humbly asks forgiveness at the hands of the man she had injured. The story ends with the reply, "'Forgive?' he said to her, scorning her for the first and only time in their history. 'Does a man forgive the hand that sets him free, the voice that re-creates him? choose some better word—my wife!'"

* "Marcella," by Mrs. Humphrey Ward. (Smith, Elder, and Co. 1894.)

### Appointments.

The Kendal Hospital.—Miss Janet Lindsay has been appointed Matron of the Kendal Hospital. She was trained at the Royal Infirmary, Liverpool, and has been three and a half years Sister of one of the surgical wards in the institution. The Liverpool Infirmary is an excellent training-school, and this, added to the fact that Miss Lindsay holds the highest testimonials for her work there, goes to show that the Kendal Hospital Committee have made a wise selection. We congratulate Miss Lindsay, and wish her all success in her new position.

### Everybody's Opinion.

[Correspondence on all subjects is invited, but we cannot in any way be responsible for the opinions expressed by our correspondents. No communications can be entertained if the name and address of the correspondent is not given, or unless one side of the paper only be written on.]

### DISTRICT NURSING.—THE OTHER SIDE OF THE QUESTION.

"A Country Practitioner" writes: Admirable as Mr. Henry Burdett's article on the advantage of a trained nurse is, a point on which we are all agreed, when means allow of one being obtained, it may be well that your readers should be presented with the reverse side of the picture, and as a member of the Dorset Health Association, one of the objects of which is to train and employ cottage nurses, a class to which Mr. Burdett seems to have a strong objection, I beg to offer a few remarks on the subject, and in addition to my own strong feeling on the matter, I am much helped in presenting my case by some excellent reasons for the support of the class of cottage nurses sent to me by one of those "impatient people" to whom Mr. Burdett alludes. In towns where a fair percentage of the population may be considered "well to do" it is not only advisable but possible to raise the necessary £70 or £80 for a trained nurse's salary; but how about the innumerable country villages where this is an absolute impossibility? Are they not to have any nursing because they cannot have the best? Is the "only well-to-do" inhabitant of the village, perhaps a struggling parson with a small income and a large family, to make himself answerable for this addition to his pecuniary responsibilities, or even the squire, who in these days of agricultural depression hardly knows how to keep his paternal acres out of the market? If between them they can manage to raise some £30 a year for the purpose of maintaining a cottage nurse it will be as much as they can reasonably do, in addition to other parochial calls. Then, too, the requirements of a hospital-trained nurse are serious. She requires to begin with, interesting or hospital cases amongst the poor, but as these are generally drafted off to the cottage or county hospital, she is left with cases of chronic ulcers of the legs, measles, influenza, women recovering from parturition, and probably some cases of paralysis and other diseases, hopeless, as regards recovery. A hospital-trained nurse is not required for cases like these, surely it is better to have several women who, as "cottage nurses," have had sufficient training to take charge of such cases, rather than one hospital-trained nurse who cannot possibly look after a district, necessarily a very wide area, if the salary of a hospital-trained nurse is to be raised in it. Are these cases to be left alone because no one but a hospital-trained nurse can make a perfect poultice, or keep a chronic ulcer of the leg clean or properly dressed? Are our weakly mothers to get up a few days after their confinement because no one but a hospital-trained nurse ought to attend or is available? Then, again, the hospital-trained nurse is as a rule, from habit, unaccustomed to muddy lanes and long walks on rainy days. She requires probably a pony or a donkey cart, or she may be content with a tricycle, unaware in her innocence that many of our country cottages cannot be reached in this manner, and that possibly a pony and riding habit might form a necessary part of her outfit, whilst her despised sister, the cottage nurse, being of a more hardy nature, would be able to reach her work on her own legs. As rural inspector of cottage nurses, a hospital-trained nurse who would make surprise visits in her district would be invaluable, and the difficulty of locomotion would be much lessened, as she could choose her time, and as her visits would not require to be so frequent she could probably secure a donkey or pony cart for the occasion, and thus would not have to add the ability to ride a pony or tricycle to her other accomplishments. She would thus raise the standard of cottage nursing immeasurably by her opportune words of advice and criticism, but then she must be careful not to invade the province of the local medical man, or she will be inevitably looked upon with disfavour and distrust. Hospital trained nurses in hospitals are undoubtedly unmixed blessings, and we all owe them immense gratitude, but in the cottages of the poor they need sometimes to be reminded that they are neither called upon to be medical students on the one hand nor charwomen on the other, though they may sometimes materially hasten the recovery of the patient by attention to domestic arrangements, for which a hospital training is not necessary, but which these cottage nurses are able to render. Let us multiply the knowledge of the simple elementary principles of nursing in every possible way—if it only suffices to dispel the courage of ignorance which now so largely prevails; let us teach our cottage nurses what they may and what they may not safely attempt, let us make sure that they realise that implicit obedience to the doctor's order is the best nursing, and then trust our medical practitioners to secure, wherever possible, the services of a hospital-trained nurse when the case is too difficult for the "cottage" one. Mr. Burdett says a successful hospital trained nurse exhibits three main qualities: intelligent obedience to the doctor's orders, love for the work, and great common sense. We endeavour to select our cottage nurses so that they too shall exhibit these qualities, and we only ask that these cottage nurses shall be looked upon as humble assistants to the hospital-trained nurse, as probationers, in fact, and be put under careful professional supervision. Let trained nurses be established in every town centre with its borough, say of 3,000 or 4,000 inhabitants, but do not deny us in our rural villages the possibility of having at least one "cottage" nurse to every parish council, and one first-class district nurse or more to every district council, and under the supervision of a county council, which latter should be drawn from the medical profession in the county. May I, in conclusion, venture to express the hope, that, as the early prognostications of the future of hospital-trained nurses have proved so fallacious, even though expressed by high authority, so in like manner fears as to the future and aims of cottage nurses, expressed by Mr. Burdett, may also prove equally fallacious.

### MISSION TO DEEP SEA FISHERMEN.

Miss Constance Delap writes: May I ask you to insert in The Hospital a short notice to the effect that I shall hold a knitting competition in October for the benefit of the Mission to Deep Sea Fishermen? All particulars will be sent to anyone forwarding a stamped and addressed envelope to me at The Parsonage, Valencia, Co. Kerry.

### Where to Go.

The North East Hospital for Children, Hackney, &c.—An entertainment in aid of this hospital and other charities will be given at Queen's Hall, Langham Place, on May 7th at three o'clock. The programme is exceptionally good. Amongst others taking part are Madame Antoinette Sterling, Mons. Hollman, Mrs. Sarah Grand, Miss Gethen (late Sister Queen), and Mr. Vivian Herbert. Special orchestra tickets for nurses in uniform may be had at 1s. each from Mrs. Brewer, 21, George Street, Hanover Square. Applications for these should be made at once.

The Queen's Jubilee Hospital.—A bazaar in aid of this hospital will be held during the present week, closing on Saturday, at the Kensington Town Hall. Fancy, fine art, confectionery, and grocery stalls are arranged. At Lady Seymour's stall articles suitable for invalids will be displayed.

Workhouse Infirmary Nursing Association.—The annual gathering of the Mary Adelaide Nurses will be held at the galleries of the Nineteenth Century Art Society, 9, Conduit Street, on Thursday evening, May 31st, at eight o'clock. The medals will be presented to the nurses by H.R.H. the Duchess of Teck. Tickets may be had from Miss Wilson, Hon. Secretary, 6, Adam Street, Strand.

### The Book World for Women and Nurses.

[We invite Correspondence, Criticism, Enquiries, and Notes on Books likely to interest Women and Nurses. Address, Editor, The Hospital (Nurses' Book World), 428, Strand, W.C.]

A Manual of Obstetric Nursing. By Marian Humfrey. (London: Sampson Low, Marston and Co.)

This volume is certainly a very valuable addition to the literature of obstetric nursing. Not only is every little detail mentioned, but its special excellence consists in the very lucid manner in which all is explained. No book can take the place of practical experience in a hospital, but it seems as if the authoress had gone through a course for the express purpose of noting every little thing which she saw, and adding thereto an explanation. If it were not that the nurse's province, as distinct from that of the doctor, was so often explained, we should be inclined to take exception to such observations as "to send fair-haired, fair-skinned, blue eyed, and rather stout women of lymphatic temperament to Bournemouth would not be altogether wise," and "we, as obstetric nurses and sensible women distinctly decline for our patients those forms of champagne which are not wine at all, and whose shining merits (if they have any) are confined to a gorgeously labelled bottle and the amount of noise and froth that accompanies their escape from that dubious receptacle." In her preface the authoress claims as a special merit for her book that it is written by a woman instead of by a man. The difficulty which a woman often has of expressing her thoughts in a few words may perhaps account for the above and many similar passages. Granting the great advantage of such a book being written by a women, we think a male friend might have reduced the size of the book considerably, without in the least detracting from its merits. With the exception of this fault, the arrangement of the chapters and the advice given are beyond praise.

Lady Perfecta. By Perez Galdos. Translated from the Spanish by Mary Wharton. (London: Fisher Unwin, 1894).

Whatever interest this story may possess in the language it was originally written in, has quite disappeared in the art-less hands of the translator. So astonishing, indeed, is the English in some parts as to be almost unintelligible without the aid of Johnson's Dictionary, and in one instance even that fails to elucidate matters. The present demand in England for foreign tales is so great, that a good deal of the translated literature offered to the public is such as would not be tolerated if written in English; but we feel that the line must be drawn somewhere, and that "Lady Perfecta" lies on the wrong side of the limit. It is a tale of true love under Spanish skies—but there the romance ends, for the reader's attention is entirely engrossed by the machinations of those who will not allow the true love to run smooth.

Lady Perfecta and her brother arrange a match between their children, and the story opens with the arrival of Pepe Rey at Villa Horrenda on a visit to his aunt, Lady Perfecta, and his cousin Rosarita. The cousins fall in love at first sight, though by the description of Pepe Rey, Rosarita would seem to have been more moderate in her demands for a romantic lover than the proverbial Spanish maiden. This is part of his description in characteristic English, "He was not a great talker; only by hearing insecure ideas and inconstant criticism was he propelled to verbosity." The story is so vague that we are not certain whether Lady Perfecta was playing a double game with her brother, or whether she changed her mind about the marriage. The reader sees very little of Rosarita, as the plottings of her mother against Pepe Rey monopolise the whole story, whereas had she employed quite simple measures she would probably have attained her end without bringing about the tragic and violent death of Pepe, and the development of hereditary mania on the part of Rosarita. The following postscript is supplied by the author, he feeling, possibly, that his readers might well search wonderingly for the *motif* of his novel:

"We are now able to decide whether those persons who seemed good were really so."

But the weary reader is content to leave the decision in the hands of the author and translator.

Mental Nursing, or Lectures for Asylum Attendants. By William Harding, M.D. With many illustrations. Second edition. Enlarged and revised. (London: The Scientific Press (Limited), 428, Strand, W.C. 1894. 2s. 6d.)

The fact that this manual has already reached its second edition is good evidence of its merit, and of the fact that it supplies a felt want. It is admirably adapted to the requirements of the asylum attendant. Its language is clear and concise, and the illustrations are excellent. Chapter I. gives a general survey of the anatomy and physiology of the human body, conveyed in a simple and easily understood form. Chapter II. contains practical hints on casualties of common occurrence in asylum life, calling for prompt attention on the part of the attendant. Chapter III. gives an excellent description of the structure and functions of the nervous system. The author tersely says "the mind is not a thing apart from the body. Intelligence is as truly the work of the brain as the movement of the fingers is the result of the contraction of the muscles of the forearm." There is much in this chapter that will be read with advantage and pleasure. Succeeding chapters give concise descriptions of the various forms of mental disorder with which the asylum attendant is brought into contact—delusions, hallucinations, states of exaltation, states of depression, delusional insanity, epilepsy, general paralysis, puerperal insanity, hysteria, idiocy, &c. Other important topics are fully considered under the headings of Ventilation, Warmth, Cleanliness and Order, Food, Entertainments, The Charge Nurse, The Night Nurse, The Sick Room—some veritably golden rules are here set forth. The last chapter gives some excellent advice on the management of the insane in private houses. This manual, beginning from the very foundation, and presenting in a convenient and compact form much useful information, is calculated effectually to assist those desirous of acquiring proficiency in asylum work. Evidently the author is well acquainted with the needs of the class for whom he writes, and he is to be congratulated on the success which has attended his efforts to produce a work which will command a wide circle of readers who will appreciate its value and utility.

### MAGAZINES FOR THE MONTH.

The Humanitarian, as usual, contains much that is of interest to us all. In "Law and Common Sense," we find an account afforded us by the editor of the recent action of "Martin v. The Trustees of the British Museum." Mrs. Victoria Woodhull Martin explained in the April number of her magazine that some such explanation was forthcoming. "Women and Gambling," by Mrs. Aubrey Richardson, draws a sad picture of what is herein described as the greatest of our national sins. The writer of this article, however, comments, with justice we think, on the lessening of the evil in the present day. "When 'female labour,'" she writes, "shall no longer be so cheap as to oust the male from its rightful sphere, working women will be less tempted to increase their small earnings by that process of rash speculation which is ever induced by a low exchequer. At present the infinite littleness of women's wage not only precludes the possibility of thrift, but promotes the insiduous practice of gambling."

